# Impaired task-dependent cerebral cortex oxygenation in Glut1 deficiency

**DOI:** 10.3389/fnins.2026.1838150

**Published:** 2026-06-25

**Authors:** Kosar Khaksari, Chad Blackshear, Ana Moreno Chaza, Sasha Mari Santiago, Wei-Liang Chen, Adrian Avila, Sharon Primeaux, Juan M. Pascual, Andrea Gropman

**Affiliations:** 1 Center for Experimental Neurotherapeutics, St. Jude Children’s Research Hospital, Memphis, TN, United States; 2 Department of Statistics, St. Jude Children’s Research Hospital, Memphis, TN, United States; 3 Center for Neuroscience and Behavioral Medicine, Children’s National Medical Center, Washington, DC, United States; 4 Wellesley College, Wellesley, MA, United States; 5 Rare Brain Disorders Program, UT Southwestern Medical Center, Dallas, TX, United States; 6 Division of Child Neurology, Departments of Pediatrics, Neurology, Neuroscience and Philosophy, Weill Cornell Medicine and Cornell University, New York, NY, United States

**Keywords:** brain dysfunction, functional near infrared spectroscopy, fNIRS, Glut1 deficiency, rare disease

## Abstract

**Introduction:**

Neuronal activation increases regional glucose utilization and requires coordinated increases in tissue oxygenation. While glucose uptake is regulated by cell type-specific transporter expression, oxygen diffuses more broadly across brain tissue, linking cerebral blood flow to measurable changes in oxygenation using functional near-infrared spectroscopy (fNIRS). In glucose transporter type 1 deficiency (G1D), reduced brain glucose and glycogen levels suggest limited substrate availability to support neural activity.

**Methods:**

Individuals with G1D and age-matched controls underwent fNIRS while performing standardized cognitive tasks. Task-evoked changes in oxygenated and deoxygenated hemoglobin were recorded to quantify cortical activation and regional oxygenation responses.

**Results:**

We hypothesized that task-dependent metabolic responses are constrained in G1D. Consistent with this hypothesis, fNIRS measurements demonstrated reduced cortical oxygenation responses in individuals with G1D compared with controls.

**Discussion:**

These findings support an altered neuroenergetic response to neural activation in G1D, in which cognitive performance is preserved despite limited substrate availability and attenuated oxygenation responses, consistent with adaptive or compensatory mechanisms.

## Introduction

Glucose transporter type 1 (Glut1) deficiency (G1D) is caused by haploinsufficiency variants in the SLC2A1 gene, resulting in impaired glucose transport across the blood-brain barrier and reduced cerebral glucose availability (Seidner et al., 1998; Rajasekaran et al., 2022; Wang et al., 2023; De Giorgis and Veggiotti, 2013). Because glucose is the primary energy substrate for the brain, impaired transport can lead to a wide spectrum of neurological; manifestations, including epilepsy, cognitive impairment, dyskinesia, ataxia, and movement disorders (Pascual and Ronen, 2015; Brockmann et al., 2001). Despite the clinical variability of G1D, genotype-phenotype correlations remain poorly understood and the mechanisms underlying disease and functional brain impairment are not fully categorized.

Current biomarkers of G1D include hypoglycorrhachia in the setting of normal blood glucose level (Klepper et al., 2020; Leen et al., 2014). While neuroimaging studies have contributed to understanding brain involvement in G1D, conventional MRI and CT findings are often normal or nonspecific (Pascual et al., 2004; Klepper and Leiendecker, 2007). As a result, there remains a need for functional biomarkers capable of detecting alterations in cerebral physiology associated with impaired glucose metabolism.

Functional near-infrared spectroscopy (fNIRS) offers a noninvasive and portable method for investigating cortical hemodynamic responses during cognitive activation (Zinos et al., 2024). In the context of G1D, fNIRS can be used to monitor brain activation patterns associated with cognitive functions such as memory, attention, and executive function (Khaksari et al., 2022). fNIRS measures relative changes in the oxygenated and deoxygenated hemoglobin (HbO and HbR). By evaluating these changes to cognitive tasks, fNIRS provides an indirect measure of brain activity and neurovascular responses. Compared with functional MRI (fMRI), fNIRS is more tolerant to motion, quicker, and easier to implement in pediatric and neurologically impaired populations, making it particularly suitable for individuals with G1D. This is particularly useful for capturing real-time brain function in naturalistic settings, allowing for more ecologically valid assessments compared to traditional methods like fMRI, which often require participants to remain still in a scanner (Cui et al., 2012; Gallagher et al., 2023).

Given the metabolic nature of G1D, altered cerebral oxygenation responses during cognitive tasks may reflect abnormalities in brain energy utilization or neurovascular coupling. We previously observed reduced basal cerebral oxygen consumption in individuals with G1D (Pascual et al., 2014). However, task-related cortical hemodynamic responses in this population have not been well characterized.

The primary objective of this study was to use fNIRS to investigate cortical hemodynamic responses during cognitive activation in individuals with G1D compared with healthy controls. We hypothesized that individuals with G1D would demonstrate altered regional oxygenation responses despite comparable behavioral performance, reflecting underlying differences in cerebral metabolic function.

That being said, fNIRS has several limitations that should be considered when interpreting findings in individuals with G1D. In addition to cohort-related constraints, such as small sample size, sex imbalance and potential selection bias, methodological limitations inherent to fNIRS including data quality considerations and signal-related constraints are addressed in the section “Limitations.”

## Materials and methods

### Participants and main goal of the study

The study protocol was approved by the Institutional Review Board (IRB) at University of Texas Southwestern (UTSW) Medical Center (Study# STU2022-0493). All procedures complied with the Declaration of Helsinki. Written informed consent was obtained from participants. Participants for this study were recruited at the 2024 Glut1 Deficiency Summit held in Dallas, Texas, in June 2024 in collaboration with UTSW.

A total of 10 participants with G1D (21.64 ± 8.39 years, 6 females) and 7 control participants (18.85 ± 6.2 years, all females) were enrolled. We were unable to include data from 4 of these participants. One control was unable to complete the experiment due to personal preferences. Among the individuals with G1D, 2 were excluded because they did not understand the task due to developmental immaturity, rendering their data unusable. Additionally, the data from 1 other G1D participant was excluded due to poor signal acquisition quality related to dense hair, which compromised fNIRS signal acquisition. As a result, seven participants with G1D (20.63 ± 8.73 years, 4 females) and a comparison group included six neurotypical control participants (18.33 ± 6.6 years, all females) who completed the same set of cognitive tasks were included in the final analysis. This allowed us to evaluate both lateralized (left vs. right hemisphere) and regional differences in brain activation between individuals with the disorder and healthy controls.

### fNIRS device

**FIGURE 1:**
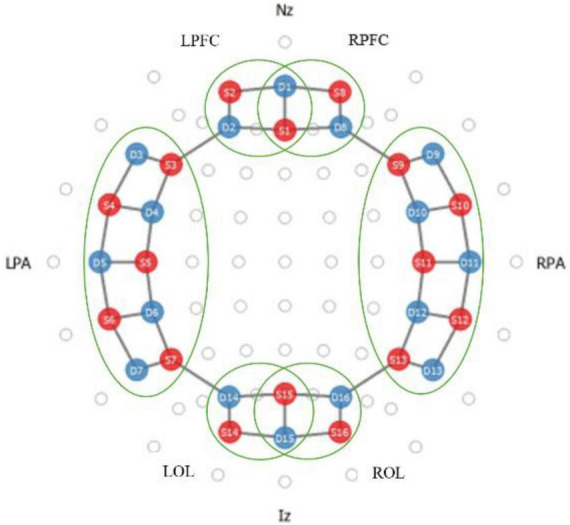
16 sources × 16 detectors cap configuration used to cover 44 fNIRS channels. Channels were grouped into six ROIs: LPFC, RPFC, LPA, RPA, LOL, and ROL.

Functional near infrared spectroscopy data were acquired using the NIRSport2 system (NIRx Medical Technologies, USA), a continuous-wave, portable neuroimaging device. A 16 × 16 configuration (16 sources and 16 detectors) was used, yielding 44 measurement channels and providing coverage across the prefrontal, parietal, and occipital cortical regions. The optode layout was based on a custom-designed configuration tailored to the regions of interest (ROIs) in this study (Figure 1). The system employs dual-wavelength light sources at approximately 760 and 850 nm to measure changes in HbO and HbR. Optical signals are detected using high-sensitivity photodiodes, and data were recorded using the Aurora acquisition software with the default sampling frequency (5.2 Hz). The device is lightweight and supports mobile data collection, giving us the ability to travel to the G1D symposium for data collection.

### ROIs and fNIRS cap configuration

The analysis focused on six predefined brain regions across the prefrontal, parietal, and occipital lobes. The selection of these regions was based on their functional relevance to cognitive and sensory processes known to be affected in G1D. The prefrontal cortex (PFC) is critically involved in executive functions and working memory, which are commonly impaired in G1D due to altered cerebral energy metabolism. The parietal area (PA) contributes to attentional control and visuospatial integration, functions that rely on distributed cortical networks with high metabolic demand. The occipital lobe (OL) was included to capture visual processing-related activity, as visual and visuospatial networks may also be affected in conditions involving altered glucose transport. Together, these regions allow assessment of both higher-order cognitive networks and primary sensory processing systems that are metabolically sensitive in G1D.

A cap configuration was designed using NIRSite (NIRx) to cover these ROIs in G1D population. NIRSite employs the EEG 10–20 system as a reference framework for montage design. Based on this system, channels were grouped into six regions of interest: left and right PFC (LPFC, RPFC), left and right PA (LPA, RPA), and left and right OL (LOL, ROL), with each region defined by averaging the signals across the corresponding channels (Figure 1).

For each region, we assessed hemodynamic responses, specifically changes in levels during all six cognitive tasks outlined in the protocol. By examining activation patterns within and across regions, as well as hemispheric asymmetries, we aimed to better characterize the neural differences associated with the disorder and identify potential biomarkers of functional impairment.

### Task: N-back

Participants aged 8 years and older completed an N-back memory task developed and utilized in our laboratory. The N-back task is a cognitive assessment designed to evaluate an individual’s working memory capacity (Owen et al., 2005). This task can be administered using either auditory or visual stimuli, and in our study, both modalities were incorporated. Stimuli were presented either as a recorded letter or a letter displayed on a computer screen. The primary goal of the task is for the participant to determine whether the current stimulus (e.g., a letter or number) matches one that was presented with a specified number of positions earlier, where this number is denoted by “N” in the term “N-back.” As the value of “N” increases, the task difficulty also escalates.

The N-back task in this experiment was created using PsychoPy software (Peirce et al., 2019) and consisted of three difficulty levels: 0-back, 1-back, and 2-back, alongside two modalities: visual and auditory, with letters serving as the stimuli. The task was divided into six trials (3 difficulty levels × 2 modalities), each lasting 150 s, leading to a total task duration of 17 min. Each trial was divided into three blocks, totaling 18 blocks throughout the experiment. Each block had a duration of 40 s, with a 10-s rest period following each block.

**FIGURE 2:**
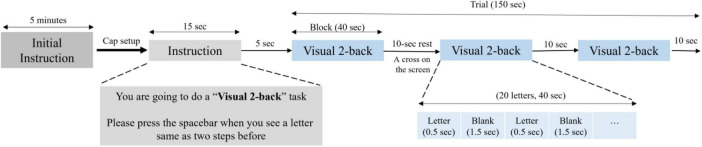
The N-back design: visual 2-back trial. The experiment consisted of 6 trials for a total time of 17 min.

In each block, 20 letters were presented either visually on the screen or auditorily via a speaker, each for 0.5 s. Following each stimulus, a 1.5-s blank interval occurred, during which either the screen remained blank, or there was silence. Prior to starting the task, participants received instructions on how to complete the N-back task, delivered through a PowerPoint presentation that included examples. Figure 2 illustrates the design of the N-back task for the visual 2-back trial. Throughout the task, continuous recordings of brain hemodynamics were obtained.

### fNIRS experimental setup

Participants were seated in a chair facing a monitor that displayed the visual version of the N-back task. A speaker placed nearby delivered the auditory version of the task. A keyboard was positioned in front of each participant for recording responses. Before beginning the experiment, the task was explained using a PowerPoint presentation that included illustrative examples. Participants then completed a practice session consisting of three mock trials to familiarize themselves with the task structure and response format. Following this, the actual task session was initiated.

The six N-back trials were randomized to prevent anticipation. Before each task began, a description was displayed on the screen, so participants knew which task to perform.

### fNIRS data analysis

Data analysis was carried out in three key stages: preprocessing, processing, and postprocessing. Preprocessing was completed using Homer3 (Huppert et al., 2009), with the following parameters: a high-pass filter cutoff at 0 Hz, a low-pass filter cutoff at 0.03 Hz to reduce high-frequency physiological artifacts and improve signal-to-noise ratio in the presence of systemic and motion-related noise in G1D patient population, and partial pathlength factors (PPFs) set to 1.0 for both wavelengths [corresponding to a commonly assumed differential pathlength factor (DPF) of approximately 6 for adult cortex in the near-infrared range]. Each subject’s dataset, including all available channels, was manually reviewed in Homer3 to assess overall signal quality.

In fNIRS, changes in HbO and HbR are estimated using the modified Beer–Lambert law (mBLL). As described by Huppert et al. (2009), both DPFs and PPFs are incorporated into the mBLL as scaling terms to account for the increased optical pathlength in scattering tissue, and their values depend on subject-specific anatomical and optical properties. Because these factors are not directly measured and can introduce uncertainty in absolute quantification, many fNIRS studies, including ours, focus on relative changes in hemoglobin concentrations. Under this framework, the PPF acts as a multiplicative scaling constant; therefore, using the default value of 1 in Homer3 does not affect the temporal dynamics or statistical comparisons of the signals, while avoiding assumptions about wavelength- or subject-specific pathlengths.

Following preprocessing, data were exported to MATLAB R2017b for further analysis, including motion artifact correction and baseline adjustment. A combination of Wavelet filtering and principal component analysis (PCA) was used to correct for noise. The Wavelet method was applied to address time-varying, frequency-specific noise such as motion and residual cardiac signals, while PCA was used to reduce spatially and globally distributed noise, such as systemic physiological signals and extracerebral contributions. Baseline correction was implemented by subtracting the average signal during the baseline period from the task-related signal, helping to reduce contamination from superficial tissue layers.

All hemoglobin signals of HbO, HbR, and HbT were considered and analyzed. A signal processing expert conducted quality control and confirmed that motion correction and baseline adjustment were uniformly applied across all signal types. For inter- and intrasubject comparisons, six defined cortical ROIs were considered by clustering anatomically corresponding fNIRS channels (see Figure 1).

In the N-back paradigm, 0-back condition was used as a low-load control (baseline) condition rather than a true resting baseline. This choice was motivated by the need to maintain consistent task engagement across conditions while minimizing working memory demands. The 0-back task requires minimal cognitive load, as it involves simple target detection rather than active maintenance of information, and is therefore commonly used in n-back paradigms as a reference condition. Importantly, using an active baseline is particularly relevant in fNIRS studies, as it helps control for differences in attention, arousal, and task engagement that may otherwise confound comparisons with higher-load conditions (1-back and 2-back). In the context of GlD, this approach also ensures that all participants remain behaviorally engaged throughout the measurement period, reducing variability due to inattention or fatigue.

A total of six task conditions were analyzed, defined by time markers captured during data acquisition. These conditions included 0-back, 1-back, and 2-back tasks, each administered in both auditory and visual formats. The analysis aimed to examine variations in cortical activation associated with increasing cognitive load, as well as to explore differences in neural response between auditory and visual modalities within each task level.

### N-back PsychoPy data analysis

Behavioral performance on the N-back task, programmed in PsychoPy, was evaluated using two key metrics: correct responses and response time. For each N-back condition (0-back, 1-back, 2-back), a correct response was defined as a spacebar press corresponding to the target stimulus for that level. The total number of correct responses was tallied for each condition, while response time was measured as the interval between stimulus presentation and the participant’s keypress. These values were aggregated across trials to obtain average correct response and response time measures, enabling comparison of cognitive load and performance stability across different task demands and modalities (auditory vs. visual).

### Statistical analysis

All analyses were performed with software (Stata version 18.0; StataCorp, College Station, TX). The primary variable of interest was the presence of G1D. The primary outcomes of interest were the trial-averaged hemoglobin concentrations (HbO, HbR, and HbT). Time series plots were prepared for each task, group, and ROI combination. We fit a three-level nested linear mixed-effects model, observations nested within task nested within participant, with fully interacted fixed effects for disease presence, task, trial, hemisphere, and region. Models were stratified by Hb concentration measured and group. Results were presented as marginal effects from each model using forest plots and heatmaps to facilitate visual comparison. Additionally, N-Back timings were presented in a time series plot with cumulative counts of correct answers during the course of testing with local polynomial smooth plots of participants within groups with 95% confidence intervals. While we present our findings as exploratory, multiple comparisons controlling for Benjamini-Hochberg false discovery rate (FDR = 0.05) were utilized to present supported findings.

## Results

### fNIRS results

**FIGURE 3:**
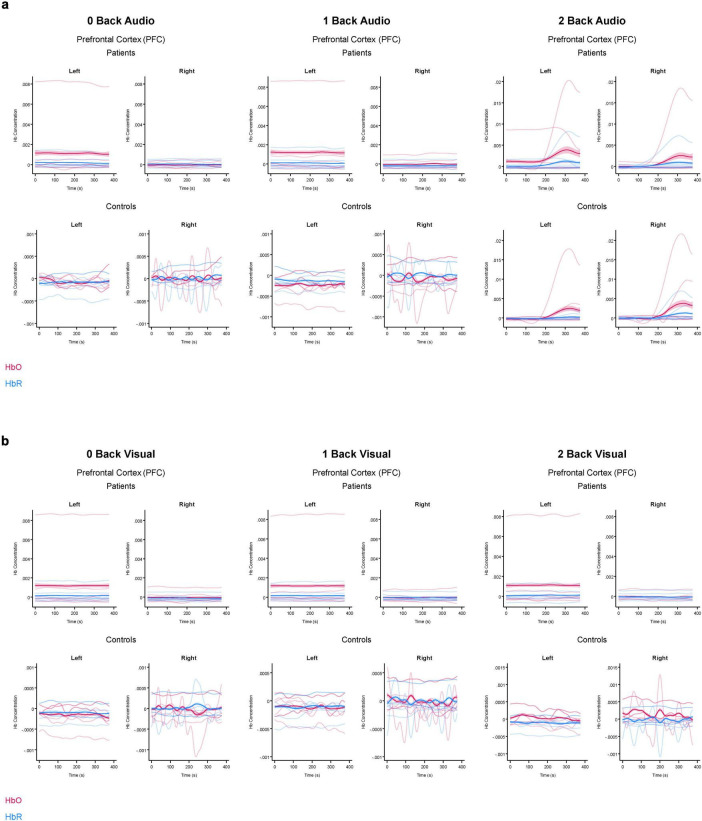
**(a)** Changes in the concentration of HbO (red) and HbR (blue) during the three tasks (0back Audio, 1back Audio, 2back Audio) in the LPFC, RPFC brain regions for patients (top) and controls (bottom). Raw measurements of participants taken during each task (lighter lines). Local polynomial smooth plots with 95% confidence intervals (darker lines). Data for the other brain regions is provided in Appendix 1. **(b)** Changes in the concentration of HbO (red) and HbR (blue) during the three tasks (0back Visual, 1back Visual, 2back Visual) in the LPFC, RPFC brain regions for patients (top) and controls (bottom). Raw measurements of participants taken during each task (lighter lines). Local polynomial smooth plots with 95% confidence intervals (darker lines). Data for the other brain regions is provided in Appendix 2.

We included 7 patients with G1D and 6 control participants in our final analysis. Changes in the concentration of HbO and HbR are plotted and considered for the comparison between patients and controls for the 6 defined ROIs during the 6 defined tasks of 0, 1, 2-back audio and visual. Figure 3 shows these changes on the LPFC and RPFC for the N-back tasks: audio (Figure 3a) and visual (Figure 3b). Please refer to Appendices 1, 2 for the other 4 ROIs.

Clinical meaningfulness in fNIRS studies is not defined by statistical significance alone, but by whether observed hemodynamic differences are large enough to reflect altered brain physiology and correlate with functional impairment (Minimal Clinically Important Differences, MCID) (Copay et al., 2007). In rare disorders like G1D, a moderate to large Hedges’ g effect size is often considered clinically meaningful because sample sizes are typically small, physiological abnormalities may be subtle, and imaging biomarkers remain exploratory rather than diagnostic. Common interpretation thresholds for Hedges’ g are: g ≈ 0.2 indicating a small difference, g ≈ 0.5 indicating a moderate difference, and g ≥ 0.8 indicating a large difference that is more likely to reflect clinically relevant physiological alterations.

Given the exploratory nature of fNIRS biomarkers in rare neurological disorders and the limited sample size, interpretation was not based solely on statistical significance. Consistent with prior exploratory fNIRS studies comparing patient and control populations, emphasis was also placed on effect size estimates to evaluate the magnitude and potential physiological relevance of observed group differences, even when *p*-values did not reach conventional significance thresholds (Chung et al., 2021; Memmini et al., 2020). As a result, both *p*-values and Hedges’ g values are presented below to provide complementary information regarding statistical trends and effect magnitude.

Forest plots and accompanying summary tables were included below to illustrate the distribution and magnitude of effect sizes across experimental conditions and ROIs. These visualizations provide a complementary representation of the quantitative findings and facilitate interpretation of group differences beyond statistical significance alone.

Additionally, heatmaps and accompanying summary tables were generated to illustrate spatiotemporal patterns of the three hemoglobin species across the six predefined brain regions over all subsets of the auditory and visual task paradigm for both patients and controls. These visualizations provide a complementary representation of hemodynamic activation patterns and facilitate qualitative comparison between groups across experimental conditions.

**FIGURE 4:**
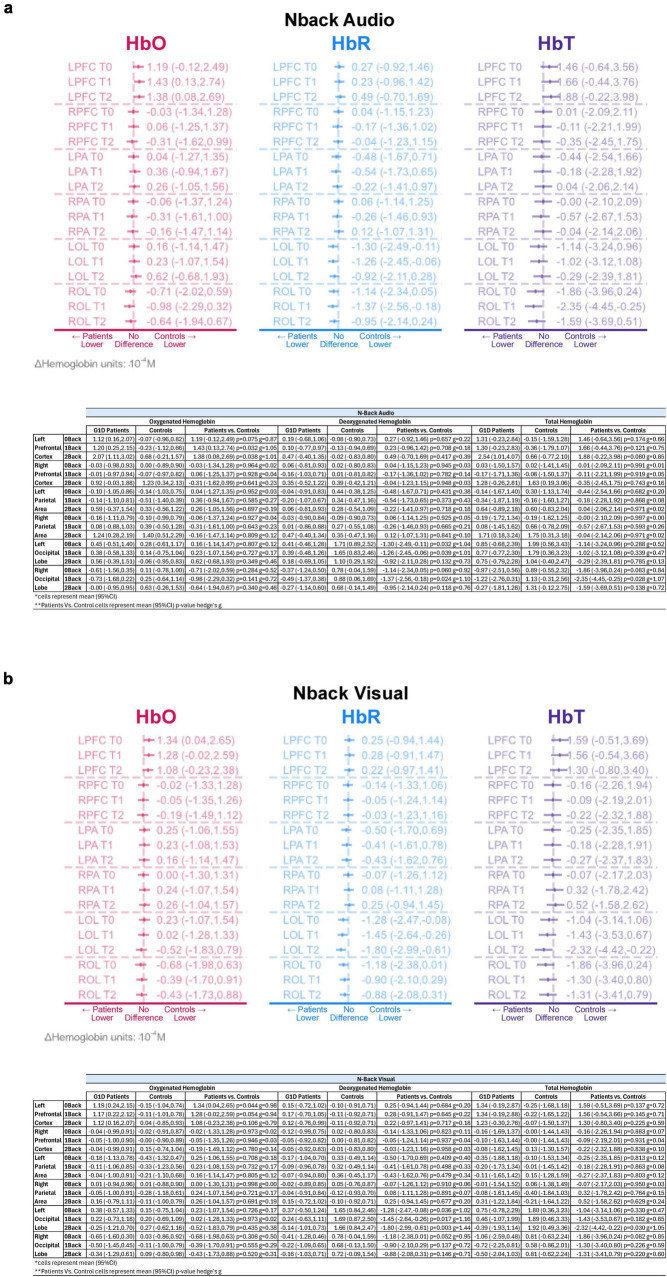
**(a)** Marginal means with 95% confidence intervals of HbO, HbR and HbT for the three audio tasks (0-back [T0], 1-back [T1], 2-back [T2]) across three brain regions (PFC, PA, OL) and two hemispheres (right and left). **(b)** Marginal means with 95% confidence intervals of HbO, HbR and HbT for the three visual tasks (0-back [T0], 1-back [T1], 2-back [T2]) across three brain regions (PFC, PA, OL) and two hemispheres (right and left).

**FIGURE 5:**
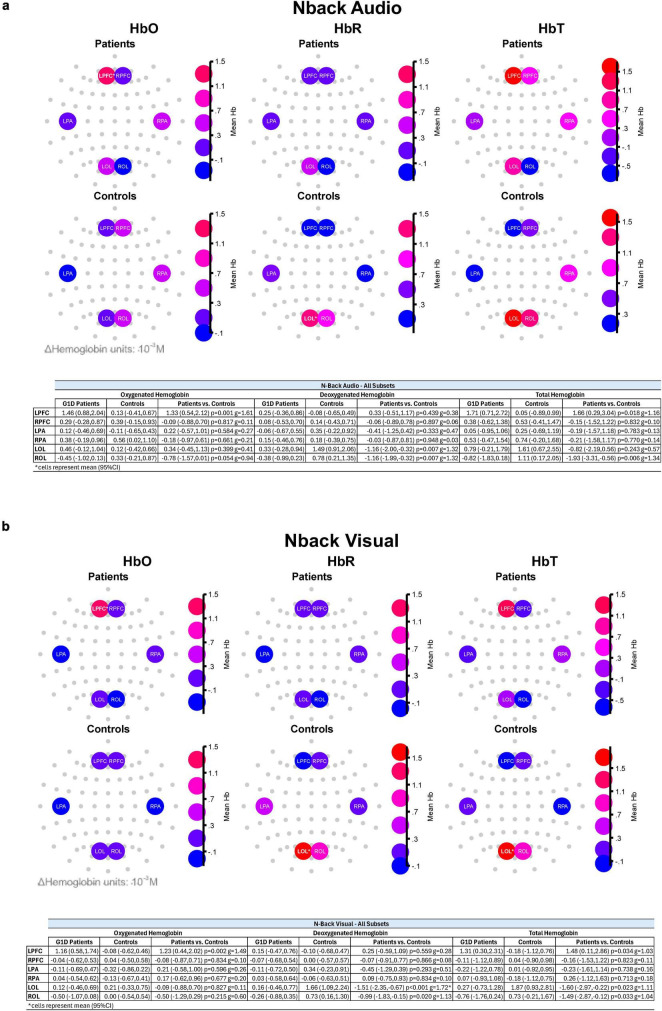
**(a)** Marginal means of HbO, HbR, and HbT for the six defined brain regions for patients (top panels) controls (bottom panels) across all subsets of the audio task paradigm. **(b)** Marginal means of HbO, HbR, and HbT for the six defined brain regions for patients (top panels) controls (bottom panels) across all subsets of the visual task paradigm.

The forest plot (Figure 4) shows overall similar activation patterns between patients and healthy controls, with the most notable activation observed in HbO within the LPFC. Changes in HbR are observed in the OL during both auditory and visual tasks. The heatmap analysis (Figure 5), which represents averaged activation across trials, further shows differential activation patterns between groups. Notably, healthy controls demonstrated a modest increase in HbR within the OL. In patients, increased HbO is observed in the LPFC, while no comparable increase is observed in the RPFC. This hemispheric asymmetry may reflect a combination of task-related, physiological, and methodological factors and is discussed further below.

### N-back PsychoPy results

**FIGURE 6:**
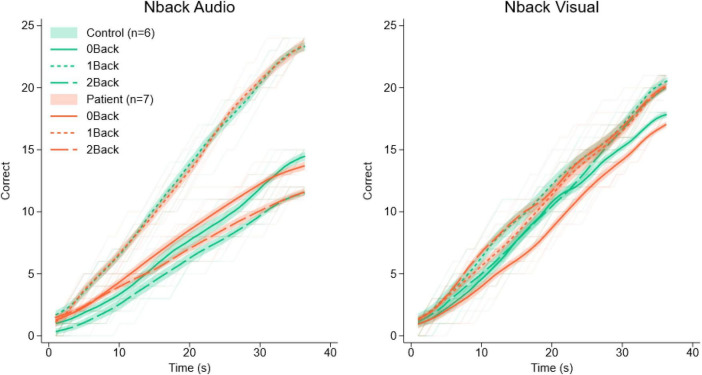
Testing performance over time. Raw measurements of timing of correct responses taken over time during each task (lighter lines). Local polynomial smooth plots with 95% confidence intervals (darker lines). These results are based on 7 individuals with G1D and 6 individuals without G1D.

Behavioral performance plots illustrating task accuracy and reaction time for patients and controls across the experimental conditions are presented below (Figure 6). Overall behavioral performance was generally comparable between groups despite observed differences in task-related hemodynamic responses measured by fNIRS.

N-back audio testing appears to have more variability in timing and overall performance over the tasks than N-back visual testing.

Because the primary objective of this exploratory study was to characterize hemodynamic response patterns measured by fNIRS, behavioral variables were not included as covariates in the statistical analysis. Additionally, the limited sample size reduced the feasibility of performing more complex multivariate analyses incorporating behavioral performance measures. Future studies with larger cohorts may further evaluate the relationship between behavioral performance and cortical hemodynamic response.

## Discussion

We have characterized, to our knowledge for the first time, the regional oxygenation of the G1D brain in response to oxygen-consuming tasks. Our previous work yielded an estimation of reduced brain glucose abundance and its regionality. Fluorodeoxyglucose positron emission tomography (FDG-PET) revealed reduced overall glucose signal at rest in a regional pattern (Pascual et al., 2002), whereas fMRI corroborated this pattern from the perspective of the restring blood oxygen level dependence signal (Rajasekaran et al., 2022). Oxygen cerebral metabolic rate (CMRO) estimates using phase-contrast MRI and T2-Relaxation Under Spin Tagging (TRUST) MRI measurements also illustrated reductions in several subjects (Pascual et al., 2014). However, these observations shed little light on task related activities due to several limitations: FDG-PET reports signal accumulation (influx minus efflux) over several minutes, fMRI of task related activity is impractical in G1D subjects due to limitations with fMRI task following, and our TRUST-MRI measurements were not sufficiently time and region-resolved. fNIRS circumvents these limitations while providing only a differential rather than absolute measure of oxygenation.

Similar alterations in task-evoked oxygenation have been reported in other neurodevelopmental and metabolic disorders, including attention-deficit/hyperactivity disorder, autism spectrum disorder, and mitochondrial encephalopathies, where blunted or delayed HbO responses have been associated with impaired neurovascular coupling, altered mitochondrial oxidative capacity, or compensatory metabolic adaptations. However, the mechanisms underlying these changes remain incompletely understood. In prior studies, the magnitude of these alterations has sometimes varied with disease severity and cognitive load, suggesting a potential relationship between underlying metabolic constraints and functional activation. We acknowledge this as a limitation of the approach and emphasize that future studies incorporating complementary metabolic measurements will be important to better understand the relationship between substrate utilization and functional activation in G1D. Thus, this should be interpreted as a testable hypothesis rather than a demonstrated mechanism.

These observations nevertheless highlight the value of fNIRS as a noninvasive and developmentally tolerant method for probing neurovascular function, with particular promise for longitudinal monitoring and treatment evaluation. Importantly, normal brain development involves age-related changes in task-evoked oxygenation responses, highlighting the need to consider developmental stages when interpreting fNIRS data in pediatric populations.

While these interpretations are informed by observed effect-size patterns, they were not consistently supported by statistically significant group differences after correction for multiple comparisons and should therefore be considered exploratory.

In this context, the G1D results may suggest a relatively reduced oxygenation response during sustained cognitive activity. Because the tasks completed were standardized and compared with age matched controls, these findings raise the possibility that G1D individuals may perform similar tasks while exhibiting lower task-evoked cerebral oxygenation responses. Several interpretations could account for this pattern, including excess consumption of non-oxidative metabolic substrate or relatively reduced cerebral oxidative metabolism. Evidence for the altered substrate utilization has been inferred in G1D rodent brain (Marin-Valencia et al., 2012; Rajasekaran et al., 2022), although its relevance to human brain metabolism remains uncertain. In individuals with G1D who are not in sustained therapeutic ketosis and who exhibit otherwise normal systemic carbohydrate metabolism, ketone availability alone would not be expected to quantitatively replace glucose utilization. Thus, the present findings may reflect altered metabolic adaptation or reduced oxidative energy demands during task performance, although these possibilities remain speculative. Likewise, sufficient cerebral ketogenesis to fully sustain metabolic demands appears unlikely based on prior rodent studies (Rajasekaran et al., 2022).

### fNIRS interpretation

In G1D, even relatively small fNIRS differences may matter biologically because the disorder directly affects cerebral glucose transport and neurovascular metabolism. Therefore, consistent reduction in HbO response and delayed hemodynamic recovery may reflect subtle cortical hypometabolism, despite the relatively modest absolute numeric differences and effect sizes observed.

Given the absence of MCID thresholds for fNIRS findings in G1D, potentially meaningful differences may be inferred when group level hemodynamic abnormalities demonstrate at least small-to-moderate effect sizes and are broadly consistent with known clinical manifestations such as cognitive slowing, executive dysfunction, fatigue, or impaired cerebral energy metabolism (Rahman et al., 2020).

### Hemispheric asymmetry

Hemispheric asymmetry, with increased activation in the LPFC but not the RPFC, may be explained by a combination of factors.

From a task perspective, the cognitive paradigm employed (N-back) is known to preferentially engage left-lateralized prefrontal networks, particularly those associated with working memory, verbal processing, and executive function. This may lead to greater recruitment of the LPFC compared to the RPFC, especially in populations relying on compensatory cognitive strategies.

From a physiological standpoint, individuals with Glut1 may exhibit altered neurovascular coupling or asymmetric functional reorganization, which could contribute to lateralized hemodynamic responses. Additionally, variability in disease severity, cognitive capacity, and task engagement across participants may further accentuate these differences.

We also acknowledge that methodological factors, including signal quality, probe placement, and the relatively small sample size, may contribute to the observed asymmetry. Future studies with larger cohorts and more detailed task characterization will be necessary to determine whether this lateralization reflects a consistent neurophysiological pattern or task-specific effect.

### Integration of behavioral performance and fNIRS findings

Behavioral performance measures (accuracy and reaction time) were generally comparable between patients and controls across tasks, suggesting preserved overt task performance despite differences in cortical hemodynamic responses. In contrast, fNIRS results demonstrated group differences in task-related HbO and HbR patterns across selected regions, particularly when examined using effect size estimates. The combination of comparable behavioral output with divergent hemodynamic responses may indicate differences in the underlying neural processes supporting task execution, rather than differences in task performance *per se*. However, given the exploratory nature of this study and the absence of statistically robust behavioral–neuroimaging correlations, interpretations regarding compensatory or efficiency-related mechanisms remain tentative. Future studies with larger cohorts and formal brain–behavior correlation analyses will be required to clarify the relationship between cognitive performance and cortical activation patterns in this population.

## Limitations

### Cohort limitations

Small sample size and sex imbalance: the relatively small sample size and sex imbalance reflect the rarity of G1D and the consequent challenges in participant recruitment. As a rare neurological disorder, available patient populations are limited, and strict inclusion criteria further constrain enrollment. Therefore, the present cohort represents a feasible sample within the context of an infrequent clinical population. These factors should be considered when interpreting the findings.

Selection bias: the patient participants recruited from the G1D conference tended to be older, which may be attributed to several factors. Conferences of this nature are more accessible to individuals and families who have received a confirmed diagnosis, are medically stable, and are able to travel and participate in structured activities. Younger children may not yet be diagnosed, as G1D can present with nonspecific or mild early symptoms that delay recognition. Additionally, families of newly diagnosed or undiagnosed children may not yet be connected to the G1D community or aware of available support groups and advocacy organizations. These barriers can reduce participation from younger or more recently identified individuals, leading to a study sample skewed toward older participants who are more likely to be engaged with the community and better positioned to attend such events.

Two participants with more severe phenotypes were excluded due to inability to complete the fNIRS protocol or generate analyzable data. This introduces a selection bias toward higher-functioning individuals and limits the generalizability of our findings to the broader population of individuals with G1D, particularly those with more severe neurocognitive impairment. Future studies incorporating adaptive paradigms or resting-state approaches may help capture a more representative spectrum of disease severity. As such, the observed patterns likely represent a “best-case” functional phenotype rather than the full neurobiological spectrum of the disorder.

### fNIRS limitations

Data Quality (General fNIRS Consideration): Before each experimental session, fNIRS signal quality was optimized to ensure reliable data acquisition. When suboptimal signals were detected, optode placement was adjusted to improve scalp contact and minimize obstructions, such as hair. Factors like skin melanin, hair color, and hair density can affect light transmission, and certain hairstyles (e.g., braids or ponytails) may interfere with cap placement. Participants with ponytails were asked to temporarily remove them, whereas tightly braided hair that could not be undone occasionally prevented participation. In this study, one participant was excluded due to hair density, but all other participants included yielded adequate data quality. Motion artifacts were noted in some younger participants, but these were mitigated using standard artifact correction methods to ensure the integrity of the final dataset (Yücel et al., 2025).

Although fNIRS is relatively tolerant to motion and well-suited for pediatric and cognitively impaired populations, motion artifacts and extracerebral signals can still impact data quality. Variability in participant characteristics including age, cognitive ability, and disease severity as well as differences in hair and skin characteristics and probe placement, may further contribute to inter-subject variability (Huang et al., 2022).

Methodological limitations in G1D: fNIRS measures hemodynamic responses primarily in cortical regions and does not capture activity in deeper brain structures that may also be affected in G1D. Second, the signals reflect changes in HbO and HbR rather than direct measures of neuronal activity or glucose metabolism and are therefore influenced by factors such as cerebral blood flow and neurovascular coupling. This is particularly relevant in G1D, where metabolic alterations may affect the relationship between neural activity and hemodynamic responses (Ferrari and Quaresima, 2012).

### Limited statistical power

Several observed associations did not remain statistically significant following correction for multiple comparisons, likely reflecting limited statistical power related to the modest sample size. Nevertheless, the observed Hedges’ g effect sizes were non-trivial and generally consistent in direction, suggesting that the underlying hemodynamic patterns may still hold potential physiological or clinical relevance. Therefore, these findings should be interpreted cautiously as exploratory and hypothesis-generating rather than definitive, warranting validation in larger and adequately powered future studies.

## Data Availability

The raw data supporting the conclusions of this article will be made available by the authors, without undue reservation.

## Appendix 1

These figures demonstrate the changes in the concentration of HbO (red) and HbR (blue) during the three tasks (0back Audio, 1back Audio, 2back Audio) in the LPA, RPA, LOL and ROL brain regions for patients (top row) and controls (bottom row). Raw measurements of participants taken during each task (lighter lines). Local polynomial smooth plots with 95% confidence intervals (darker lines). For the LPFC and RPFC regions, refer to Figure 3a.

## Appendix 2

These figures demonstrate the changes in the concentration of HbO (red) and HbR (blue) during the three tasks (0back Visual, 1back Visual, 2back Visual) in the LPA, RPA, LOL and ROL brain regions for patients (top row) and controls (bottom row). Raw measurements of participants taken during each task (lighter lines). Local polynomial smooth plots with 95% confidence intervals (darker lines). For the LPFC and RPFC regions, refer to Figure 3b.
